# The comparison of perioperative outcomes of robot-assisted and open partial nephrectomy: a systematic review and meta-analysis

**DOI:** 10.1186/s12957-016-0971-9

**Published:** 2016-08-22

**Authors:** Zhonghua Shen, Linguo Xie, Wanqin Xie, Hailong Hu, Tao Chen, Chen Xing, Xiaoteng Liu, Hao Xu, Yu Zhang, Zhouliang Wu, Dawei Tian, Changli Wu

**Affiliations:** 1 Department of Urology, The Second Hospital of Tianjin Medical University, Pingjiang Road 23, Hexi District Tianjin, 300211 China; 2 Tianjin Key Laboratory of Urology, Tianjin Institute of Urology, the Second Hospital of Tianjin Medical University, Tianjin, 300211 China; 3 Key Laboratory of Genetics and Birth Health of Hunan Province, The Family Planning Research Institute of Hunan Province, Changsha, Hunan 410126 China

**Keywords:** Robot-assisted partial nephrectomy, Open partial nephrectomy, Renal tumor, Meta-analysis

## Abstract

**Background:**

Robot-assisted partial nephrectomy (RAPN) has been widely used worldwide, to determine whether RAPN is a safe and effective alternative to open partial nephrectomy (OPN) via the comparison of RANP and OPN.

**Methods:**

A comprehensive literature search was performed within the databases including PubMed, Cochrane Library, and Embase updated on 30 September 2015. Summary data with their corresponding 95 % confidence intervals (CIs) were calculated using a random effects or fixed effects model. Heterogeneity and publication bias were also evaluated.

**Results:**

A total of 16 comparative studies including 3024 cases were used for this meta-analysis. There are no significant differences in the demographic characteristic between the two groups, but the age was lower and the tumor size was smaller for the RAPN group. RAPN had a longer operative time and warm ischemia time but which showed less estimated blood loss, hospital stay, and perioperative complications. No differences existed in the margin status, the change of glomerular filtration rate, transfusion rate, and conversion rate between the two groups. There was no significant publication bias.

**Conclusions:**

RAPN offered a lower rate of perioperative complications, less estimated blood loss, and shorter length of hospital stay than OPN, suggesting that RAPN can be an effective alternative to OPN. Well-designed prospective randomized controlled trials will be helpful in validating our findings.

## Background

Renal tumor has become a significant threat to the public health, with a high incidence rate of 12.6/10,000 and 6.7/10,000 for men in the developed and less developed regions, respectively [1]. Partial nephrectomy and radical nephrectomy are the two surgical options to deal with renal tumor. Because partial nephrectomy may have less renal function impairment, better overall survival, and equivalent oncological survival compared to radical nephrectomy, the European Association of Urology recommended partial nephrectomy, when feasible, as a gold standard treatment for patients presenting with small renal carcinoma [2].

There are three different ways to perform partial nephrectomy, namely the open partial nephrectomy (OPN), laparoscopic partial nephrectomy (LPN), and robot-assisted partial nephrectomy (RAPN). Among these available nephron-sparing surgery options, OPN is the most extensively studied and has been demonstrated with comparable oncological efficacy, less incidence of chronic kidney disease within a 10-year follow-up, less cardiovascular morbidity, and overall mortality compared to radical nephrectomy [3, 4]. LPN has been shown to offer better cosmetic results, less postoperative pain, shorter length of hospital stay, and faster postoperative recovery than OPN, but the steep learning curve leads to its limited diffusion in the high-volume reference centers and application to the small and less complex tumors [5]. Although a few studies have compared the perioperative outcomes of OPN and RAPN, their results are inconsistent. Han et al. [6] and Alemozaffar et al. [7] found that RAPN caused longer operative time than OPN, whereas other studies [8–10] showed the opposite finding. Regarding the complication rate, the comparison between RAPN and OPN in two studies [11, 12] also yielded with conflicting results.

Based on these observations, we believe there is a necessity for a systematic review to compare the perioperative outcomes of RAPN and OPN with the most updated data and draw a more accurate conclusion.

## Methods

### Publication search

The systematic review followed the Cochrane review guidelines. A comprehensive literature search using the combinations of key words “open,” “robotic/robot-assisted,” and “partial nephrectomy” was done within the electronic databases PubMed, Cochrane Library, and Embase updated on 30 September 2015. The publications in English rather than other languages were collected. The computer search was supplemented with manual searches within the reference lists of all retrieved studies, review articles, and conference abstracts. Two authors (Chen Tao and Hu Hailong) reviewed the titles and abstracts of all items returned by the search engine to assess their relevance to this meta-analysis independently. When the two authors could not make an agreement on certain items, Xie Linguo served as a third reviewer to look into the full text and make a decision that whether the publication is qualified for this study.

### Inclusion and exclusion criteria

All available randomized controlled trials (RCTs) and retrospective comparative studies (cohort or case-control studies) that compared RAPN with OPN were included. Studies of comparison of RAPN, LPN, and OPN were also included as long as the data for RAPN and OPN could be extracted and had at least one of the outcomes mentioned in the paper. Editorials, review articles, and animal experimental studies were excluded. When multiple reports described the same population, the most recent or complete report was used.

### Data extraction

Data associated with the included studies were extracted and summarized by two authors (Chen Tao and XieLinguo) independently. A senior author (Hu Hailong) was responsible for resolving disagreements pertaining to data extraction if there were any. The extracted information contained the demographic data (age, gender, body mass index, tumor size, and the location of tumor), the information about the source of controls, study design, and sample size of the study population, and the perioperative outcomes including operating time (OT), warm ischemia time (WIT), blood loss, length of hospital stay (HS), positive surgical margins (PSM) rate, change of glomerular filtration rate (eGFR), perioperative complications rate, transfusion rate, and conversion rate (RPN converted to laparoscopic or open partial nephrectomy, RPN or OPN converted to radical nephrectomy). The day of follow-up, the number of tumor recurrence, metastasis, and death were also extracted.

### Statistical analysis and quality assessment

The random effects model was used when significant heterogeneity existed among the included studies as assessed by the inconsistency index (*I*^2^ > 50 %) and the chi-square test with significance heterogeneity (*p* < 0.10); otherwise, the fixed effects model was used [13]. The level of evidence of the included studies was rated according to the criteria provided by the Centre for Evidence-Based Medicine in Oxford, UK (CEBM home page, available online http://www.cebm.net/index.aspx?o=1025, accessed on September 5, 2014). The modified Newcastle-Ottawa scale was used to assess the quality of the retrospective study [14] (The Newcastle-Ottawa Scale (NOS) for assessing the quality of non-randomized studies in meta-analyses. http://www.ohri.ca/programs/clinical_epidemiology/oxford.asp, accessed on September 15, 2014). This evaluation system consists of three factors: assessment of outcome, comparability of the study groups, and patient selection. A score ranging from 0 to 9 (represented by 0 to 9 stars) was provided as the result of the assessment for each study, except for RCTs (Table 1). The studies which scored by 6 or more stars were considered to be of high quality. All the meta-analyses were performed using Review Manager 5.2 (Cochrane Collaboration, Oxford, UK). Weighted mean difference (WMD) and odds ratio (OR) were used to compare continuous and dichotomous variables, respectively. All results were reported with 95 % CIs. All reported *p* values were two sides, the value of *p* < 0.05 was considered to indicate statistical significance, and publication bias was assessed by Funnel plots. The Clavien-Dindo score system was used to stratified postoperative complication for subgroup analysis [15].

**Table 1:** Characteristics of the included studies

Study	Level of evidence	Design	Matching^a^	Follow-up (month)^f^ RAPN/OPN	No. of centers	Quality score^g^
Wu et al. [10]	IIIb	R	1,2,3,4,6,8	12 (6–24)/12 (6–24)	Single	☆☆☆☆☆☆☆
Vittori et al. [11]	II	PN	1,2,3,4,7,8	Perioperative	Multicenter	☆☆☆☆☆☆
Masson-Lecomte et al. [16]	IIIb	RP	1,2,3,4,6,8	19 (6–30)/32 (12–40)	Single	☆☆☆☆☆☆☆
Ficarra et al. [17]	IIIb	R	1,2,5,6,7,8	Perioperative	Multicenter	☆☆☆☆☆☆☆
Kim et al. [24]	IV	R	1,2,3,4,5,6	NA/NA	NA	☆☆☆☆
Lucas et al. [18]	IIIb	R	1,2,3,6,7,8,9	9.4 ± 7.6/21.1 ± 18.8	Single	☆☆☆☆☆☆
Han et al. [6]	IIIb	RP	1,2, 4,5,6,7,8	Perioperative	Single	☆☆☆☆☆☆☆
Oh et al. [19]	IIIb	R	1,2,3,4,5,6,7, 8,9	Perioperative	Single	☆☆☆☆☆☆☆☆
Lee et al. [9]	IV	R	1,2,5,6,8	NA/NA	Single	☆☆☆☆☆
Alemozaffar et al. [7]	IIIb	R	2,3,4,5,6,7	Perioperative	Single	☆☆☆☆☆☆
Simhan et al. [12]	IIIb	RP	1,2,3,4,6,7	17.1 ± 9.2/23.9 ± 20.5^b^; 21.3 ± 13.3/19.8 ± 11.1^c^	Single	☆☆☆☆☆☆
Zargar et al. [20]	IIIb	R	1,2,3,5,6,7	7.8 (18.7)/14 (14.5)^d^; 4 (18)/19.6 (29.7)^e^	Multicenter	☆☆☆☆☆☆
Boylu et al. [8]	II	PN	1,2,3,5,6,8	33 ± 13/37 ± 20	Single	☆☆☆☆☆☆
Mano et al. [22]	IIIb	R	1,2,3,4,5,6,7	Perioperative	Single	☆☆☆☆☆☆
Miyake et al. [23]	IIIb	R	1,2,3,4,5,6,7,8	1/1	Single	☆☆☆☆☆☆
Webb et al. [21]	IIIb	R	1,2,5,6,8	NA/NA	Single	☆☆☆☆☆

*R* retrospective, *RP* retrospective analysis, prospective data collecting, *PN* prospective non-randomized design, *NA* not available

^a^Matching: 1 = age; 2 = gender; 3 = body mass index; 4 = American Society of Anesthesiologists score; 5 = tumor laterality; 6 = tumor size; 7 = nephrometry score (RENAL or PADUA); 8 = pre-op eGFR; 9 = single surgeon

^b^Moderate nephrometry group (NS 7–9)

^c^High nephrometry group (NS 10–12)

^d^Simple tumors (RENAL score 4–8)

^e^Complex tumors (RENAL score 9–12)

^f^The follow-up time was reported in the form of “median (inter-quartile range)” or “mean ± standard deviation” or not recorded

^g^Modified Newcastle-Ottawa Scale (NOS)

## Results

### Literature search and characteristics of the included studies

**Fig. 1:**
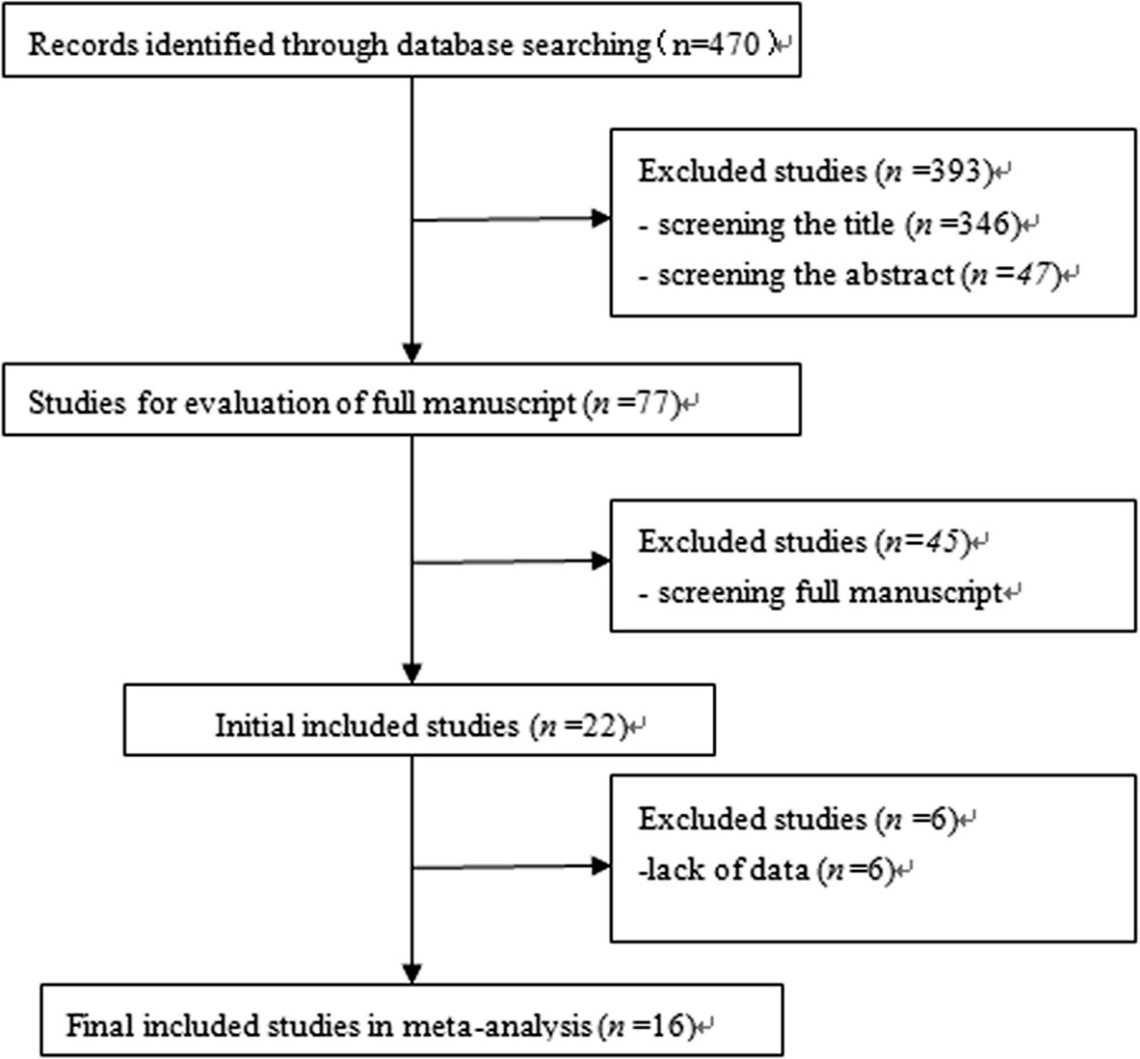
Flow diagram of the comprehensive literature search

The results for each step of the literature search are shown in Fig. 1. Eventually, a total of 16 publications fulfilled the predefined inclusion criteria, including 15 full-text articles [6–12, 16–23] and 1 conference abstract [24]. Two of the 13 studies compared the outcomes of patient groups stratified by RENAL score [12, 20]. Thus, in total, there were 18 independent study populations included in this meta-analysis. Of the 16 studies, 4 studies conducted the comparison of perioperative outcomes of RAPN, LPN, and OPN [6, 7, 18, 21], 12 studies documented their single center’s experience with RAPN versus OPN [6–10, 12, 16, 18, 19, 21–23], 3 studies [11, 17, 20] involved the multicenter collaboration, and 10 studies [6, 7, 11, 12, 17–20, 22, 23] matched the two approach with renal tumor nephrometry score (RENAL or PADUA). Among the included studies, there was 2 prospective nonrandomized comparative study (level of evidence: II) [8, 11], 3 studies retrospective analysis but prospective data collecting (level of evidence: III) [6, 12, 16], 9 retrospective studies compared contemporary of patients (level of evidence: III) [7, 10, 17–23], 1 retrospective study used a historical series as the control (level of evidence: IV) [9], and 1 retrospective study but a conference abstract (level of evidence: IV) [24].

Our literature search found no randomized and blinded studies available for this meta-analysis. Seven studies [8, 10, 12, 16, 18, 20, 23] declared the duration of follow-up for both groups. Table 1 summarizes the characteristics of the included studies.

### Demographic characteristics of the study populations

This meta-analysis involved 3024 cases (1103 cases for RAPN and 1921 cases for OPN) (Table 2). There are no significant differences in the gender, patients of benign: malignant, and body mass index (BMI) of patients between RAPN and OPN, but the age was lower (WMD, −1.52 years; 95 % CI, −2.53 to −0.51; *p* = 0.003) and the tumor size was smaller for RAPN group (WMD, −0.46 cm; 95 % CI, −0.66 to −0.26; *p* < 0.01).

**Table 2:** Demographic and perioperative results of RAPN vs OPEN

Study (OPN*/*RAPN)	Patients	Males	Age mean ± SD (year)	Benign: malignant	Tumor size mean ± SD size (cm)	BMI mean ± SD (kg/m^2^)
Wu et al. [10]	94/51	62/35	NA/NA	NA:NA/NA:NA	NA/NA	NA/NA
Vittori et al. [11]	198/105	123/69	63.8 ± 12.4/62.3 ± 11.6	42:156/14:91	3.5 ± 1.8/2.8 ± 1.5	NA/NA
Masson-Lecomte et al. [16]	58/42	40/22	60.8 ± 11.2/61.7 ± 10.9	8:33/6:52	3.1 ± 1.2/2.8 ± 1.4	26.5 ± 5.6/26.9 ± 4.2
Ficarra et al. [17]	200/200	131/121	62.4 ± 11.8/62.4 ± 10.6	NA:NA/NA:NA	NA/NA	NA/NA/
Kim et al. [24]	83/67	56/48	56.2 ± 14.7/51.5 ± 11.9	NA:NA/NA:NA	2.6 ± 1.7/2.3 ± 1.1	24.8 ± 3.3/24.2 ± 3.0
Lucas et al. [18]	54/27	38/19	NA/NA	10:44/10:17	NA/NA	NA/NA
Han et al. [6]	354/147	270/108	55.3 ± 12.4/52.5 ± 11.9	NA:NA/NA:NA	2.80 ± 1.35/2.58 ± 1.13	24.5 ± 3.0/25.56 ± 3.2
Oh et al. [19]	100/100	69/70	54.59 ± 13.40/54.27 ± 11.52	NA:NA/NA:NA	2.59 ± 1.35/2.52 ± 1.26	25.14 ± 2.73/25.48 ± 3.47
Lee et al. [9]	234/69	164/50	54.36 ± 12.77/53.48 ± 11.85	NA:NA/NA:NA	2.58 ± 1.40/2.37 ± 1.26	24.49 ± 2.80/25.50 ± 3.20
Alemozaffar et al. [7]	25/25	19/15	61.9 ± 10.1/55.9 ± 11.7	0:25/0:25	3.3 ± 1.4/2.5 ± 1.0	30.1 ± 5.9/27.5 ± 3.8
Simhan et al. 1 2012 [12]	136/81	89/43	58.7 ± 11.2/56.6 ± 13.1	2:136/3:81	4.1 ± 2.3/3.2 ± 1.8	30.0 ± 7.0/30.7 ± 6.7
Simhan et al. 2 2012 [12]	54/10	28/6	59.4 ± 10.8/56.1 ± 10.7	2:54/1:10	5.4 ± 3.8/3.7 ± 2.5	30.9 ± 6.8/30.7 ± 3.5
Zargar et al. 1 2014 [20]	33/30	NA/NA	NA/NA	NA:NA/NA:NA	NA/NA	NA/NA
Zargar et al. 2 2014 [20]	52/10	NA/NA	NA/NA 7	NA:NA/NA:NA	NA/NA	NA/NA
Boylu et al. [8]	20/46	14/29	56 ± 13.5/54 ± 12	6:14/9:37	4.04 ± 2.08/3.56 ± 1.36	27.5 ± 3.3/28.7 ± 3.1
Mano et al. [22]	190/63	132/46	NA/NA	NA:NA/NA:NA	NA/NA	29 (25–31.5)/29.1 (26.6–31.5)^a^
Miyake et al. [23]	15/16	10/14	64.2 ± 12.2/63.3 ± 13.2	NA:NA/NA:NA	3.2 ± 0.9/3.0 ± 0.9	24.4 ± 5.1/24.9 ± 4.2
Webb et al. [21]	21/14	14/6	53.6 ± 10.05/60.5 ± 13.45	2:19/2:12	4.22 ± 1.34/2.99 ± 1.10	NA/NA

^a^Median (25th–75th)

### Comparison of perioperative outcomes of RAPN and OPN

Table 3 summarizes the perioperative outcomes of RAPN and OPN.

**Table 3:** Perioperative outcomes of RAPN versus OPN

Study (OPN/RAPN)	OT (min)	WIT (min)	MBL (ml)	Positive margins (%)	HS (day)	eGFR change (ml/min per 1.73 m^2^)	Transfusion rate (%)	Conversion rate (%)
Wu et al. [10]	NA/NA	NA/NA	NA/NA	0 (0.0)/0 (0.0)	NA/NA	NA/NA	4 (4.3)/3 (5.9)	1 (1.1)/0 (0.0)
Vittori et al. [11]	123 ± 43/168 ± 56	18.7 ± 8.1/18.2 ± 7	230 ± 208/125 ± 128	11 (5.6)/6 (5.7)	NA/NA	NA/NA	NA/NA	2 (1.0)/1 (0.9)
Masson-Lecomte et al. [16]	128.4 ± 50.5/134.8 ± 35.3	17.1 ± 5.9/17.5 ± 7.8	414.7 ± 367.5/142.9 ± 225.9	4 (6.9)/1 (2.4)	6.8 ± 3.5/3.8 ± 1.1	NA/NA	NA/NA	NA/NA
Ficarra et al. [17]	NA/NA	NA/NA	NA/NA	9 (4.5)/9 (4.5)	NA/NA	−16.6 ± 18.1/−16.4 ± 22.9	20 (10.0)/21 (10.5)	NA/NA
Kim et al. [24]	126.8 ± 42.7/196.9 ± 50.1	27.8 ± 9.7/31.3 ± 9.5	356.7 ± 269.2/296.8 ± 246.8	1 (1.2)/2 (3.0)	4.6 ± 2.1/2.9 ± 1.4	−0.49 ± 17.5/−2.54 ± 16.01	13 (15.7)/5 (7.5)	7 (8.4)/4 (5.9)
Lucas et al. [18]	NA/NA	NA/NA	NA/NA	4 (7.4)/1 (3.7)	NA/NA	NA/NA	NA/NA	0 (0.0)/1 (3.7)
Han et al. [6]	187.2 ± 43.8/162.3 ± 32.3	19.6 ± 6.7/24.7 ± 7.3	NA/NA	NA/NA	7.3 ± 2.06/5.3 ± 1.41	−4.34 ± 8.34/−2.4 ± 6.7	NA/NA	NA/NA
Oh et al. [19]	138.79 ± 40.29/182.89 ± 83.98	21.18 ± 11.29/21.86 ± 9.25	230.74 ± 159.33/212.04 ± 160.76	1 (1.0)/0 (0.0)	9.26 ± 3.22/5.41 ± 1.84	−6.19 ± 7.32/−7.53 ± 4.28	6 (6.0)/4 (4.0)	NA/NA
Lee et al. [9]	142.77 ± 47.69/192.42 ± 78.05	18.14 ± 7.16/22.99 ± 8.43	216.50 ± 165.38/228.70 ± 182.89	6 (2.6)/0 (0.0)	8.90 ± 3.11/6.20 ± 1.99	−5.25 ± 10.01/−6.11 ± 9.14	NA/NA	1 (0.4)/1 (1.4)
Alemozaffar et al. [7]	238.3 ± 119.5/231.8 ± 44.2	NA/NA	275.4 ± 170.0/178.0 ± 205.7	NA/NA	4.60 ± 1.68/2.48 ± 0.68	NA/NA	NA/NA	NA/NA
Simhan et al. 1 2012 [12]	189.5 ± 52.0/205.9 ± 52.5	NA/NA	256.5 ± 291.3/131.3 ± 127.8	1 (0.7)/3 (3.7)	5.6 ± 3.9/3.7 ± 1.6	1.5 ± 21/−2.4 ± 23.1	NA/NA	NA/NA
Simhan et al. 2 2012 [12]	197.5 ± 60.4/221.1 ± 72.5	NA/NA	330.6 ± 406.0/225.0 ± 304	3 (5.6)/0 (0.0)	6.1 ± 4.1/2.9 ± 1.4	6.1 ± 25.2/−9 ± 21.2	NA/NA	NA/NA
Zargar et al. 1 2014 [20]	185.42 ± 5 6.4/174.9 ± 61.7	NA/NA	NA/NA	3 (9.1)/2 (6.7)	NA/NA	NA/NA	5 (15.2)/6 (20.0)	NA/NA
Zargar et al. 2 2014 [20]	244.1 ± 59.3/250.8 ± 66	23.9 ± 8.1/22.7 ± 5.8	NA/NA	4 (7.7)/1 (10.0)	NA/NA	NA/NA	8 (15.4)/0 (0.0)	NA/NA
Boylu et al. [8]	152 ± 18/225 ± 58	18 ± 3.5/23 ± 7.3	417 ± 202/268 ± 303	0 (0.0)/1 (2.1)	5.4 ± 2/4.11 ± 1.5	NA/NA	2 (10.0)/5 (10.9)	NA/NA
Mano et al. [22]	128 (108–156)/154 (113–177)^b^	NA/NA	200 (100–413)/100 (25–200)^b^	NA/NA	2.47 ± 1.31/1.51 ± 0.76	NA/NA	NA/NA	NA/NA
Miyake et al. [23]	203.7 ± 55.2/263.0 ± 63.5	20.3 ± 9.1/23.0 ± 7.5(1)	653.6 ± 611.7/57.5 ± 96.9	0 (0)/0 (0)	4.8 ± 0.8/4.2 ± 0.8	10.0 ± 6.6/10.4 ± 7.0	0 (0)/0 (0)	0 (0)/0 (0)
Webb et al. [21]	NA/NA	30.69 ± 10.65^a^/28.01 ± 9.34	NA/NA	1 (4.8)/1 (7.1)	4 (3–6)^b^/3 (2–4)^b^	NA/NA	0 (0)/0 (0)	2 (9.5)/0 (0)

The data of OT, WIT, MBL, HS, and eGFR change are expressed as mean ± standard deviation. The positive margin, transfusion, and conversion are shown with the number of cases and its percentage (the value in brackets) in each study

*RAPN* robot-assisted partial nephrectomy, *OPN* open partial nephrectomy; *OT* operating time; *WIT* warm ischemia time, *MBL* mean blood loss, *HS* hospital stay, *eGFR change* change of glomerular filtration rate, *NA* not available

^a^Cold ischemia

^b^Median (25th–75th)

**Fig. 2:**
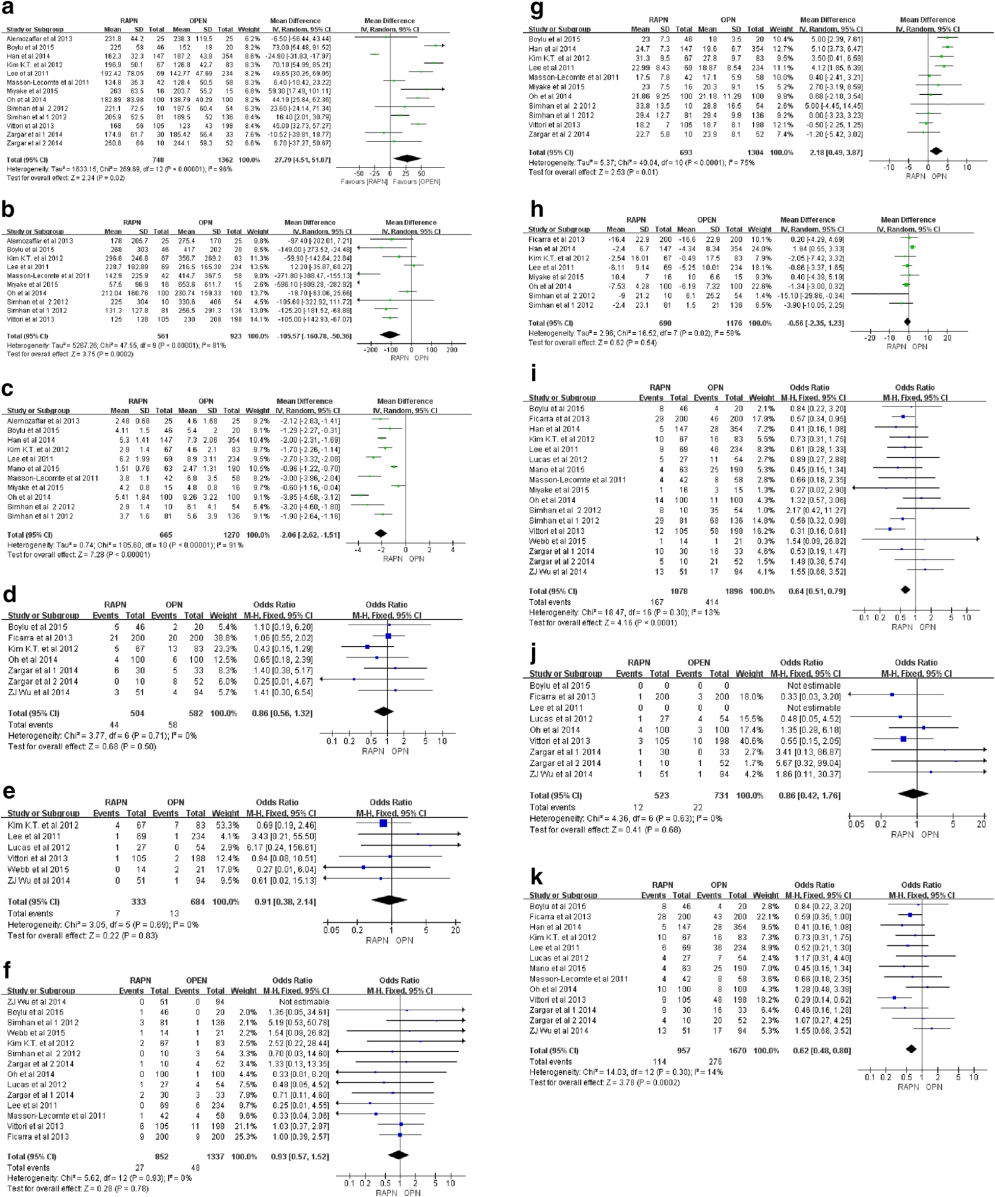
Forest plots of the perioperative outcomes of RAPN versus OPN. **a** Operative time. **b** Estimated blood loss. **c** Length of hospital stay. **d** Transfusion rate. **e** Rate of conversion to radical nephrectomy. **f** Positive margin rate. **g** Warm ischemia time. **h** Estimated GFR change. **i** Overall complication rate. **j** Intra-operative complicate rate. **k** Postoperative complication rate

The operative time was statistically shorter in the OPN group (WMD, 27.79 min; 95 % CI, 4.51 to 51.07; *p* = 0.02) (Fig. 2a), but the estimated blood loss (WMD, −105.57 ml; 95 % CI, −160.78 to −50.36; *p* = 0.0002) (Fig. 2b) was less in the RAPN group, and a significantly shorter postoperative hospital stay (WMD, −2.06 day; 95 % CI, −2.62 to −1.51; *p* < 0.001) (Fig. 2c) was found in the RAPN group. Eight studies [8, 10, 17, 19–21, 23, 24] examined the perioperative transfusion, and we found that there was no significant difference in transfusion rate between RAPN and OPN (OR, 0.86; 95 % CI, 0.56 to 1.32; *p* = 0.50) (Fig. 2d). Seven studies [9–12, 18, 21, 24] made the comparison of intraoperative conversion rate of RAPN and OPN; as a result, we found no difference in conversion rate between RAPN and OPN (OR, 0.91; 95 % CI, 0.38 to 2.14; *p* = 0.83) (Fig. 2e). Thirteen of the 16 included studies for this meta-analysis examined the margin status of surgical specimens [8–12, 16–21, 23, 24], no significant difference was observed regarding the positive margin rate between RAPN and OPN based on the data from the 11 studies involving 2220 cases (OR, 0.93; 95 % CI, 0.57 to 1.52; *p* = 0.78) (Fig. 2f). The overall warm ischemia time of RAPN was significantly longer than that of OPN (WMD, 2.18 min; 95 % CI, 0.49 to 3.87; *p* = 0.01) as supported by the pooled data from 10 studies [6, 8, 9, 11, 12, 19, 20, 23, 24] (Fig. 2g). There was no difference in the change of eGFR between RAPN and OPN (WMD, −0.56 ml/min per 1.73 m^2^; 95 % CI, −2.35 to 1.23; *p* = 0.54) (Fig. 2h).

### Comparison of complications of RAPN and OPN

Table 4 summarizes the complications of the two groups.

**Table 4:** Complication rates of RAPN versus OPN

Study (OPN/RAPN)	Patients	Overall complication (%)	Intracomplication (%)	Postcomplication (%)	Clavien1 (%)	Clavien2 (%)	Clavien3 (%)	Clavien4 (%)	Clavien5 (%)	Minor (%)	Major (%)
Wu et al. [10]	94/51	18 (19.1)/14 (25.9)	1 (1.1)/1 (2.0)	17 (18.1)/13 (25.5)	16 (17.0)/12^a^		1 (1.1)/1 (2.0)^b^			16 (17.0)/12 (23.5)	1 (1.1)/1 (2.0)
Vittori et al. [11]	198/105	58 (29.3)/12 (11.4)	10 (5.1)/3 (2.9)	48 (24.2)/9 (8.6)	3 (1.5)/1 (1.1)	21 (10.6)/4 (3.8)	15 (7.6)/1 (1.0)	3 (1.5)/0 (0.0)	NA/NA	24 (12.1)/5 (4.8)	18 (9.1)/1 (1.0)
Masson-Lecomte et al. [16]	58/42	8 (13.8)/4 (9.6)	NA/NA	8 (13.8)/4 (9.6)	0 (0.0)/2 (4.8)	6 (10.3)/2 (4.8)	2 (3.4)/0 (0.0)	NA/NA	NA/NA	6 (10.3)/4 (9.5)	2 (3.4)/0 (0.0)
Ficarra et al. [17]	200/200	46 (23.0)/29 (14.5)	3 (1.5)/1 (0.5)	43 (21.5)/28 (14.0)	34 (17.0)/19 (9.5)^a^		7 (3.5)/8 (4.0)	2 (1.0)/1 (0.5)	NA/NA	34 (17.0)/19 (9.5)	9 (4.5)/9 (4.5)
Kim et al. [24]	83/67	16 (19.3)/10 (14.9)	NA/NA	16 (19.3)/10 (14.9)	1 (1.2)/0 (0.0)	13 (15.7)/7 (10.5)	0 (0.0)/2 (3.0)	1 (1.2)/1 (1.5)	1 (1.2)/0 (0)	14 (16.9)/7 (10.4)	2 (2.4)/3 (4.5)
Lucas et al. [18]	54/27	11 (20.4)/5 (18.5)	4 (7.4)/1 (3.7)	7 (13.0)/4 (14.8)	5 (9.3)/1 (3.7)	1 (1.9)/2(7.4)	1 (1.9)/0 (0.0)	0 (0.0)/1 (3.7)	NA/NA	6 (11.1)/3 (11.1)	1 (1.9)/1 (3.7)
Han et al. [6]	354/147	28 (7.9)/5 (3.4)	NA/NA	28 (7.9)/5 (3.4)	4 (1.1)/2 (1.4)	17 (4.8)/1 (0.7)	7 (2.0)/2 (0.2)^c^		NA/NA	21 (5.9)/3 (2.0)	7 (2.0)/2 (0.2)
Oh et al. [19]	100/100	11 (11.0)/14 (14.0)	3 (3.0)/4 (4.0)	8 (8.0)/10 (10.0)	NA/NA	NA/NA	NA/NA	NA/NA	NA/NA	NA/NA	NA/NA
Lee et al. [9]	234/69	46 (19.6)/9 (13.0)	10 (4.3)/3 (4.3)	36 (15.4)/6 (8.7)	20 (8.5)/3 (4.3)	5 (2.1)/3 (4.3)	11 (4.7)/0 (0.0)	NA/NA	NA/NA	25 (10.7)/6 (8.7)	11 (4.7)/0 (0.0)
Alemozaffar et al. [7]	25/25	NA/NA	NA/NA	NA/NA	NA/NA	NA/NA	NA/NA	NA/NA	NA/NA	NA/NA	NA/NA
Simhan et al. 1 2012 [12]	136/81	68 (50.0)/29 (35.8)	NA/NA	NA/NA	NA/NA	NA/NA	NA/NA	NA/NA	NA/NA	NA/NA	NA/NA
Simhan et al. 2 2012 [12]	54/10	35 (64.8)/8 (80.0)	NA/NA	NA/NA	NA/NA	NA/NA	NA/NA	NA/NA	NA/NA	NA/NA	NA/NA
Zargar et al. 1 2014 [20]	33/30	16 (48.5)/10 (33.3)	0 (0.0)/1 (3.3)	16 (48.5)/9 (30.0)	6 (18.2)/3 (10)	3 (9.1)/4 (13.3)	3 (9.1)/2 (6.7)	4 (12.1)/0 (0.0)	NA/NA	9 (27.3)/7 (23.3)	7 (13.5)/2 (6.7)
Zargar et al. 2 2014 [20]	52/10	21 (40.4)/5 (50.0)	1 (1.9)/1 (10.0)	20 (38.5)/4 (40.0)	10 (19.2)/0 (0)	4 (7.7)/0 (0.0)	3 (5.8)/1 (10.0)	3 (5.8)/3 (30.0)	NA/NA	14 (26.9)/0 (0.0)	6 (11.5)/4 (40.0)
Boylu et al. [8]	20/46	4 (20)/8 (17.3)	0 (0.0)/0 (0.0)	4 (20)/8 (17.3)	NA/NA	NA/NA	NA/NA	NA/NA	NA/NA	NA/NA	NA/NA
Mano et al. [22]	190/63	25 (13)/4 (6)	NA/NA	25 (13)/4 (6)	14 (7)/4 (6)^a^		11 (6)/0 (0)	0 (0.0)/0 (0.0)	0 (0.0)/0 (0.0)	14 (7)/4 (6)	11 (6)/0 (0)
Miyake et al. [23]	15/16	3 (20.0)/1(8.3)	NA/NA	3 (20.0)/1(8.3)	1 (6.7)/0 (0)^a^		2 (13.3)/1 (8.3)^b^			1 (6.7)/0 (0)	2 (13.3)/1 (8.3)
Webb et al. [21]	21/14	1 (4.7)/1 (7.14)	NA/NA	NA/NA	NA/NA	NA/NA	NA/NA	NA/NA	NA/NA	NA/NA	NA/NA

*NA* not available

^a^Clavien 1 and 2 complications

^b^Clavien 3–5 complications

^c^Clavien 3 and 4 complications were merged as one group for discussion in the original data sources

The overall complication rate of RAPN was significantly lower than that of OPN (OR, 0.64; 95 % CI, 0.51 to 0.79; *p* < 0.001) by the pooled data from 15 studies [6, 8, 10–12, 16–24] (Fig. 2i). Intraoperative complication rate was available for 8 studies [8–11, 17–20], and no significant difference existed between the two groups (OR, 0.86; 95 % CI, 0.42 to 1.76; *p* = 0.68) (Fig. 2j). Postoperative complication rate was mentioned in 13 studies [6, 8–11, 16–20, 22–24], and patients from the 11 studies [6, 9–11, 16–18, 20, 22–24] were further divided into the minor (Clavien classification 1–2) complication subgroup and major (Clavien classification 3–5) complication subgroup. The pooled data favored RAPN for lower rates of overall postoperative complication (OR, 0.64; 95 % CI, 0.51 to 0.79; *p* < 0.001) (Fig. 2k), minor complication (OR, 0.62; 95 % CI, 0.46 to 0.83; *p* = 0.001), and major complication (OR, 0.57; 95 % CI, 0.36 to 0.91; *p* = 0.02) compared to OPN.

### Comparison of postoperative efficacy of RAPN and OPN

Eight studies [8–10, 12, 16, 18, 20, 21] compared the recurrence or metastasis rate of the RAPN with that of the OPN. Among the 8 studies, there were 13 recurrences, 1 metastasis, and 5 deaths in the OPN group, 756 patients were included, while there were 0 recurrence, 1 metastasis, and 2 deaths in the RAPN group including 380 patients. The ratio of tumor recurrence metastasis and death were 2.5 and 0.8 % in the OPN and RAPN, respectively. However, the data were not pooled for meta-analysis due to the different lengths of follow-up period between the studies.

### Heterogeneity and publication bias analysis

**Fig. 3:**
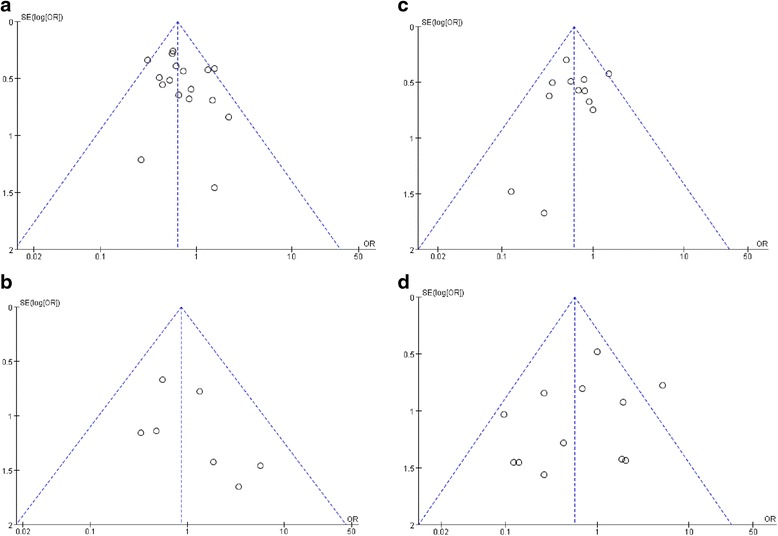
Funnel plots illustrating meta-analysis of perioperative complication rates. **a** Overall complication. **b** Intraoperative complication. **c** Minor complication. **d** Major complication. *SE* standard error, *OR* odds ratio

In this meta-analysis, the *Q*-test and the *I*^2^ index were used to evaluate the heterogeneity across studies. As shown in Fig. 2, there was no heterogeneity among the Dichotomous variable, on the contrary, there was statistically significant heterogeneity among the continuous variables. Figure 3 shows that the funnel plots of the studies included in this meta-analysis reported perioperative complication rates. Almost all studies lie inside the 95 % CIs (overall complication with *P* = 0.30; intraoperative complication with *P* = 0.63; minor complication with *P* = 0.58; major complication with *P* = 0.07), with an even distribution around the vertical, indicating no obvious reporting bias.

## Discussion

LPN and RAPN have been increasingly accepted as minimally invasive nephron-sparing surgical modalities over the past two decades. Several studies [25, 26] have made the comparison and concluded that the surgical and oncologic outcomes provided by LPN are comparable to those by OPN. However, LPN requires proficient laparoscopic skills and the steep learning curve limits its wide application in partial nephrectomy. With the rapid advances in technology, RAPN has emerged as a new option to deal with small renal tumors, but whether PARN is safe and effective to deal with renal masses, we should compare it to OPN that is the matching standard of treatment and has the robustness of data regarding surgical and oncological results [27]. To make a comparison between RAPN and OPN, Wu et al. [10] performed a meta-analysis with the data extracted from 8 studies including 3418 patients (757 patients in the robotic group and 2661 patients in the open group); however, 2 of the 8 studies compared only the cost between RAPN and OPN [28, 29], and thus only 1118 patients (359 patients in the robotic group and 759 patients in the open group) accounted for the comparison in their study.

Here, we collected the data from 16 studies [6–8, 10–12, 16–24] including 3024 cases (1103 cases for RAPN and 1921 cases for OPN) to compare the outcomes between RAPN and OPN. We notice that the tumor size of the RAPN group is smaller than that of the OPN group. This could be caused by the selection bias that RAPN is recommended to deal with small renal masses, but we can see that all of the included studies matched the two groups with tumor size except Vittori et al. [11, 22]. Nephrometry score, which derives from the systematic analysis of anatomical renal tumor characteristics, plays an important role in PN outcomes reporting because it indicates the degree of technical complexity and permits valid comparison among different cohorts. In this meta-analysis, we observed 11 studies [6, 7, 11, 12, 17–23] matched the two approaches with renal tumor nephrometry score (RENAL or PADUA). We believe that it is comparable for the two groups. RAPN has a lower age than OPN, but all of the studies included in this meta-analysis matched the two groups with age except Alemozaffar et al. [7]. Together with these, demographic characteristics of the study populations have a little impact on the outcome of the two groups, although the selection bias is existed.

The operative time was significantly longer in the RAPN group than in the OPN group, Masson-Lecomte et al. [16] found that the difference in operative time was insignificant between RAPN and OPN when “skin-to-skin” time (excluding the setup and docking time) rather than the total operating room occupation time. And we can find that the operative time in RAPN group was shorter than Wu et al.’s [10] meta-analysis, we believe that the operative time of RAPN will be shorter than OPN in the future, because RAPA enables a flexible, precise, and rapid operation. On the contrary, RAPN has better outcomes in terms of EBL when compared with the open group. In addition to this, length of hospital stay was significantly shorter in the RAPN group. Boylu et al. [8] found that the mean operation time was significantly longer and the EBL was less for the RAPN group, but they found that the mean postoperative decline of hematocrit was not statistically different between the two groups, which suggests that RAPN provided rapid convalescence, decreased hospital stay, and less blood loss when compared to the OPN group. We did not see the difference in the margin status between the two groups in our analysis either. Additionally, no significant differences in the transfusion rate and surgical conversion rate between RAPN and OPN were observed in this study, which also suggested that RAPN is comparable to OPN.

Our analysis showed that the overall WIT was higher in the RAPN group. WIT < 30 min is recommended in order to reduce renal ischemic injury [30], and a more recently published multicenter study suggests that the optimal WIT should be <20 min in order to preserve optimal renal functions [5]. Thompson et al. [31] suggest that WIT < 25 min is a safety standard for partial nephrectomy. Ideal WIT is still under debate in the current literature, but most of the authors agreed that WIT < 25 min is a safety standard for partial nephrectomy. All of the included studies in this meta-analysis meet the standard, except the Simhan et al. [12] and Kim et al. [24]. Since the amount of renal parenchyma removed rather than WIT is the determinant for the final degree of renal function preservation [32, 33], WIT is unlikely to be the limiting factor for RAPN. Despite the significantly longer WIT in the RAPN group, we found that the change of eGFR is comparable between the two groups, suggesting that RAPN and OPN apparently have the same efficacy on postoperative renal function.

We found that there was no significant difference in intraoperative complication rate between the two groups, which was inconsistent with the finding by Wu et al. [10]. We pooled the data from 7 studies associated with Clavien grade 2 complication, which requires treatment with drugs, and found that RAPN had lower chance of Clavien grade 2 complication than OPN; this can also be seen in Clavien grade 3 complication (*p* = 0.003). Only 1 study compared the Clavien grade 5 complication (death) and found that there was no difference between the two groups (*p* = 0.421). Zhang et al. [34] compared the perioperative and oncologic outcomes of localized renal tumors treated by RAPN with those treated by LPN and found no difference between them. Vittori et al. [11] demonstrated that open surgical approach is the only independent risk factor associated with Clavien grade 3–4 complications. Here, we found that there was no difference between RAPN and OPN regarding Clavien grade 3–4 complications. In the subgroup analysis of postoperative complications, minor complications and major complications frequently occurred following OPN group.

A trend was observed toward a higher failure of cancer control rate for OPN (2.5 versus 0.8 %), it is not appropriate to estimate the weighted effect with the hazard ratio of tumor recurrence and metastasis for the differences in the length of follow-up duration between the studies.

We realize that there are limitations in this meta-analysis. Firstly, all the included studies are retrospective, non-randomized comparisons, except 2 prospectively derived comparative studies. Secondly, no follow-ups of long period have been achieved for RAPN, so the data availability for tumor recurrence and metastasis were quite limited. Thirdly, all continuous variables had a big heterogeneity which may contribute to the different sample sizes, multiple surgeons with different surgical experiences, tumor complexity, and the lack of RENAL standardization between groups. In addition, almost all previous studies deal with small renal masses. This reminds us a caution of potential selection bias. As a newly emerging surgical option for the treatment of renal masses, RAPN should be compared to OPN that has robust data regarding the surgical and oncological outcomes and serves as the justification standard. Although it will be helpful to perform prospective randomized studies comparing RAPN with OPN, such studies in need of recruiting a homogeneous group of patients with renal masses are difficult to carry out under the context of real clinic.

## Conclusions

This meta-analysis reveals that RAPN results in a significantly lower rate of perioperative complications, less estimated blood loss, and shorter hospital stay, but longer operative time and estimated warm ischemia time than that of open approach. There are no differences in the margin status, transfusion rate, and conversion rate between RAPN and OPN. Thus, RAPN can be an effective alternative to OPN. Well-designed prospective randomized controlled trials will be helpful in validating our findings. With the accumulating knowledge about RAPN, LPN, and OPN, the best decisions regarding the surgical technique for organ-sparing renal tumor resection may be made under the consideration of both patient and surgeon’s preference.

## Abbreviations

RAPN: Robot-assisted partial nephrectomy
OPN: Open partial nephrectomy
BMI: Body mass index
WMD: Weighted mean difference
OR: Odds ratio
CI: Confidence interval
EAU: European Association of Urology
LPN: Laparoscopic partial nephrectomy
RCTs: Randomized controlled trials
OT: Operating time
WIT: Warm ischemia time
HS: Hospital stay
PSM: Positive surgical margins
eGFR: Glomerular filtration rate
NOS: The Newcastle-Ottawa Scale
R: Retrospective
RP: Retrospective analysis, prospective data collecting
PN: Prospective non-randomized design
EBL: Estimated blood loss
